# Classification, Diagnosis, and Prognosis of Cardiomyopathy: A Comprehensive Narrative Review

**DOI:** 10.31083/RCM36280

**Published:** 2025-06-30

**Authors:** Yubin Jin, Wenjing Che, Jiuyue Yang, Shumin Chang, Wenqi Bao, Xinyue Ren, Pengyu Yu, Aijie Hou

**Affiliations:** ^1^Department of Cardiovascular Internal Medicine, The People’s Hospital of China Medical University, The People’s Hospital of Liaoning Province, 110000 Shenyang, Liaoning, China

**Keywords:** review, classification, diagnosis, prognosis, cardiomyopathy

## Abstract

Cardiomyopathy denotes a group of heart diseases caused by structural or functional heart muscle disorders, with various genetic and non-genetic etiologies. Based on the current literature, this narrative review synthesizes key findings from available information on the classification, diagnosis, and prognosis of inherited or acquired cardiomyopathies. Following a different approach to prior systematic reviews, this study does not implement any formal inclusion or exclusion criteria or structured search strategy. However, this review does consider the evidence of influential studies, prominent cardiology guidelines, and expert consensuses to provide a comprehensive overview of recent advancements in the field. Further, explication is performed for the latest advances in genetic mutations, diagnostic imaging techniques, and therapeutic techniques. All diagnoses involve clinical presentations, imaging, and laboratory tests. Future research directions include personalized therapy, quantitative imaging techniques, and new drug treatments. This review highlights cardiomyopathy research by emphasizing the integration of precision medicine, advanced imaging, and molecular diagnostics. Future research on cardiomyopathy should include precision medicine and personalized therapies with an exhaustive integration of techniques and resources to catalyze further innovations in diagnostics and therapeutic approaches. Thus, this narrative review will provide clinicians and researchers with insight into the future of cardiomyopathy management by summarizing key developments and trends.

## 1. Introduction

Cardiomyopathy denotes an array of myocardial diseases, each characterized by structural and functional aberrations of the heart muscle. The associated conditions may result in heart failure, arrhythmias, and even sudden cardiac death in very grave cases [1, 2]. The mechanistic basis of cardiomyopathy presents genetic, acquired, or multifactorial aspects, with clinical presentation widely variable according to age, ethnic groups, genetic context, and further environmental influences [3]. The burden of cardiomyopathy is significant worldwide, hence the need for continuous innovation in diagnostics, classification methods, and treatment strategies geared towards improving patient outcomes [2].

Technological innovations have changed cardiomyopathy research in the past decades by girdering genetic screening techniques, imaging modalities, and artificial intelligence (AI)-driven diagnostic tools into disease detection and management [4]. Although technological advancements support this era of the complete naval watch, numerous gaps still remain in our knowledge, covering the classification of cardiomyopathy subtypes, development of suitable prognostic markers, and realization of precision medicine strategies [5]. This identifies a clear necessity to curate an updated synthesis of current developments wherein we carefully merge genetic and imaging-based diagnostics, paving the way for implementing new treatment approaches [6, 7].

The paper is a narrative review discussing the classification, diagnosis, and prognosis of cardiomyopathy. Unlike a systematic review, it did not adopt any predetermined approach regarding search strategy for data, predefined inclusion and exclusion criteria, or PRISMA guidelines. However, it synthesized the findings of high-impact research studies, key cardiology guidelines, and expert consensus to provide an overview of the recent developments in the research on cardiomyopathy [8]. It allows discussion of new trends, diagnostic techniques, and therapeutic strategies, which are not generally elaborated upon in systematic reviews or meta-analyses [9].

The review aims to synthesize recent advancements in cardiomyopathies in classification, diagnosis, and prognosis [10]. The focus here is to describe the classification of cardiomyopathies, differentiate between the hereditary and acquired forms, and not forget to mention differences attributed to distinct aetiologies, pathophysiological mechanisms, and clinical manifestations [11]. Genetic mutations are pivotal in hereditary cardiomyopathies, comprising hypertrophic, dilated, and restrictive subtypes [12]. Generally, acquired cardiomyopathies are occurring due to external factors such as alcohol, ischemia, or myocarditis [13]. Thus, this will improve understanding of the disease progression and provide an important foundation for governing diagnosis and management.

Advances in diagnostic modalities are essential for detecting and classifying cardiomyopathies in their early stages [14]. This review outlines the emerging role of AI in cardiology, mainly to improve automated analysis of echocardiography, cardiac magnetic resonance imaging (MRI), and computed tomography (CT) [15]. Moreover, it discusses how strain echocardiography and parametric cardiac MRI can precisely characterize myocardial anomalies [16]. Another significant change in managing cardiomyopathies is incorporating genetic testing in the diagnostic flow to identify potential candidates for targeted treatment [17]. By converging on these recent technological changes, this review translates the importance of incorporating such advanced diagnostic tools into the regular workings of clinical practice.

The discussion extends to prognostic markers and treatment options, emphasizing the shifting landscape of cardiomyopathy management. With the arrival of precision medicine, genetic and molecular profiling are increasingly being utilized to tailor treatment options to individual patients [18]. The review looks into the newer pharmacologic interventions, like myosin inhibitors, sodium-glucose cotransporter inhibitors, and gene editing therapies, that are changing implementation and management strategies [19]. Pharmacogenomics is also gaining momentum, enabling the selection of drugs most effective for each patient while minimizing adverse effects through a genetic profile-based approach.

The advancement of future studies in stratifying cardio-myopathies towards personalized and technologically driven approaches [20]. *CRISPR* and RNA-based therapies are gene-editing technologies poised to modify the disease-causing mutations, conferring the ability to possibly stop or even reverse the hereditary cardiomyopathy’s progression [21]. Further anticipated developments with artificial intelligence and machine learning will add to advanced clinical outcomes by delivering more precise diagnostics and risk stratification via inspecting complex datasets from imaging, genetic, and biomarker studies [22]. Shortly, continuous monitoring through wearable devices and remote monitoring technology should transform the care and management of patients via the continuous monitoring of cardiac function and early disease progression detection.

With these discoveries, cardiomyopathy diagnosis and treatment have progressed into modern management through genetic and molecular cardiology. The arrival of gene-editing techniques, particularly *CRISPR-Cas9* and RNA-targeted drugs, offers precision medicine in correcting the pathogenic mutations linked to inherited cardiomyopathies [23]. Novel biomarker discovery has also aided in the diagnosis and risk stratification, which includes circulating microRNAs, natriuretic peptides, and high-sensitivity troponins [24]. Biomarkers provide insights into cardiac remodeling, inflammation, and fibrosis, impacting treatment decisions and long-term prognosis [25]. At the molecular level, TGF-β mediated fibrotic pathways, oxidative stress responses, and mitochondrial dysfunction play important roles in disease progression and heart failure risk in cardiomyopathy [26]. The better understanding of the genetics and molecular aspects shall work toward targeted therapies for individualized treatment strategies [27].

This narrative review combines conspicuous trends and developments. It will aid the clinician and the researcher through the lens of cardiomyopathy evolving, aimed at bridging the existing knowledge gaps and informing future research endeavors.

## 2. Classification of Cardiomyopathy

### 2.1 Classification Based on Etiology and Clinical Manifestations

**Fig. 1.**
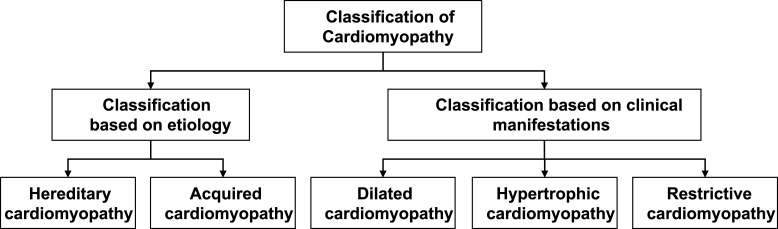
**Classification of cardiomyopathy**.

Cardiomyopathy can be classified into two categories based on etiology and three categories according to clinical manifestations (Fig. 1).

#### 2.1.1 Hereditary Cardiomyopathy

Hereditary cardiomyopathy is a term that refers to a mix of disorders resulting from genetic mutations that alter the structure or function of myocardial cells and, subsequently, result in impaired cardiac functioning. The genetic disorders may involve one or more genes and pathways, leading to cardiac muscle structural changes such as hypertrophy, dilation, or stiffness. Advances in genetic testing have recently revealed specific gene mutations- including myosin, troponin, and titin- that play a fundamental role in the pathophysiology of these entities. There are also increasing interventions from recent innovations such as artificial intelligence or AI tools to interpret genetic data to improve diagnostic precision and tailor therapeutic strategies. The main subtypes of hereditary cardiomyopathy, hypertrophic cardiomyopathy (HCM), dilated cardiomyopathy (DCM), and restrictive cardiomyopathy (RCM), present distinct clinical features and genetic backgrounds. However, genetic testing practices are highly heterogeneous across laboratories, posing a major challenge to their diagnosis and treatment [28].

#### 2.1.2 Acquired Cardiomyopathy

Acquired cardiomyopathy is a cardiac condition that arises from several external factors like infection, toxins, or some medications that could change the structure or function of the myocardium. While hereditary forms of cardiomyopathy stem from inherited mutations, acquired cardiomyopathies respond to environmental or lifestyle factors. Some of these include alcohol-induced cardiomyopathy, myocarditis, and chemotherapy-induced cardiomyopathy [22]. Complete non-invasive cardiac MRI and echocardiography imaging may greatly improve the diagnosis of acquired forms. Still, reliability and access to these techniques remain problems, particularly in low-resource settings. Management for acquired cardiomyopathy is usually directly supportive of the heart, including treating the cause of the cardiomyopathy (as in discontinuing toxic medications or addressing infections) [29].

### 2.2 Classification Based on Clinical Manifestations

#### 2.2.1 Dilated Cardiomyopathy

DCM, which involves enlargement and weakening of the heart muscles, reduces the ability of the heart to pump blood. In most cases, they may present symptoms like heart failure, arrhythmias, fatigue, dyspnea, and, in extreme cases, sudden death [4]. Both gene mutation and acquired factors make DCM probable secondary to viral infections, toxins (alcohol), and autoimmune diseases [25]. Though imaging studies like cardiac MRI and echocardiography are widely used for diagnosis, AI-enabled interpretation of imaging studies may further substantially facilitate enhanced diagnostic accuracy and interpret real-time data for guidance during treatment [5]. With the increased genetic and environmental factor variations inherent in DCM, personalized treatment approaches are increasingly emphasized.

#### 2.2.2 Hypertrophic Cardiomyopathy

HCM is characterized by extreme heart muscle thickening, most notably in the left ventricle, which may impede the heart’s ability to fill with blood. The most common defect is thought to be congenital genetic defects in genes that code for cardiac proteins such as myosin and troponin [18]. Extension in genetic testing allows for identifying persons at risk of developing HCM. This invariably leads to early diagnosis and, thus, timely intervention. Clinical symptoms, which may involve genetic testing or imaging studies, usually confirm a clinical diagnosis of HCM, with cardiac MRI and echocardiography being the most important investigations. However, the limited availability and the cost of advanced imaging in certain parts of the world may be bottlenecks. Management options, for example, involve beta-blocks and surgical options and may depend on the disease’s severity and the patient’s genetic profile [30].

#### 2.2.3 Restrictive Cardiomyopathy

Restrictive cardiomyopathy refers to a rare heart disorder that is associated with stiffening of the myocardium, which is a retardation of the heart during filling. Though other forms see a more preserved contractile function of the heart, in RCM, the ventricles fail to relax, causing diastolic dysfunction. RCM may result from some infiltrative diseases like amyloidosis sarcoidosis, and metabolic diseases [8]. Genetic testing has become more important in differentiating inherited forms of RCM, while cardiac MRI has become an important diagnostic tool for evaluating myocardial stiffness and pericardial involvement [9]. In the usual case, treatment aims at the primary cause; in advanced cases, a heart transplant is frequently the only option. The use of AI for earlier detection via better imaging modality is a developing area of research, as it provides a better evaluation of changes to the myocardial tissue [10].

### 2.3 Diagnosis of Cardiomyopathy

#### 2.3.1 Clinical Manifestations and Symptoms

The clinical features and symptoms exhibited by cardiomyopathy often vary depending on the type and severity of the disease; nonetheless, they generally include:

(1) Shortness of breath: Patients may feel short of breath during physical activity or at rest because the heart loses the ability to pump powerfully enough to maintain adequate cardiac output. In DCM, breathlessness worsens, impairing the heart’s pumping function.

(2) Fatigue and weakness: Fatigue or weakness, often explained by impaired pump function, is generally found among patients with cardiomyopathy. In RCM, this may also be due to fluid retention and low cardiac output.

(3) Angina: Some cardiomyopathy patients might have chest pain or discomfort localized in the precordial region or behind the sternum, especially in cases of HCM where there is pathological thickening of the heart muscle and blood flow is impeded.

(4) Arrhythmias: The patients will have various arrhythmias, including tachycardia and fibrillation. The risk of these arrhythmias increases with HCM and DCM and may be affiliated with specific genetic mutations through the application of genetic tests.

(5) Edema: Cardiac pumping ability is such that fluid retention in the tissues in cardiomyopathy may lead to edema. They usually occur in the lower legs, foot, abdomen, or other body areas. This is particularly true in DCM, where edema is very likely and may be worse with heart failure.

(6) Syncope and dizziness: Several patients with cardiomyopathy were experiencing syncope and dizziness due to failing cardiac output and low blood flow to the brain. Such symptoms are very important in diagnosis because they are often indicative of either ventricular arrhythmias or heart failure, which must be acted upon quickly.

(7) Chest pain: Some patients present with chest pain that can occur during exercise or physical activities. In most cases, HCM prompts the development of this type of chest pain, most commonly caused by the thickening of the heart muscle, which decreases blood flow.

Specific symptoms will differ according to the type and severity of the disease. With the advancement of genetic testing and imaging modalities such as cardiac MRI and cardiac magnetic resonance imaging (CMR), diagnosis and prognosis of a particular subtype of cardiomyopathy can be carried out earlier to help in management customization approaches. Automating echocardiographic analyses through AI-assisted imaging techniques helps facilitate earlier diagnoses, which are valuable in determining disease severity or trajectories for more tailored biochemical intervention that improves symptoms.

Thus, various disease manifestations need to be assessed when diagnosing and treating cardiomyopathy. Genetic analysis and AI-assisted imaging could be undertaken in a much more clinically significant context thanks to new and relatively powerful tests. These tests allow for more precise diagnoses and tailored treatment plans that address natural history, symptom presentation, and genetic etiological triggering.

2.3.1.1 Advanced Imaging Modalities

Recent advances in cardiac imaging have greatly improved the recognition and prognostication of cardiomyopathy in early clinical manifestations. Strain echocardiography, and particularly global longitudinal strain (GLS), provides a sensitive measure of myocardial deformation, enabling the detection of systolic dysfunction at an early stage even prior to the development of changes in ejection fraction [11]. This method yields some promising results in tracking the diagnosis and progression of heart disease in hypertrophic and dilated cardiomyopathy with a predominance of subclinical disease and adding to risk stratification [12].

Parametric mapping in magnetic resonance imaging allows for non-invasive tissue characterization and provides insights into myocardial edema and fibrosis. Unlike late gadolinium enhancement, T1 and T2 mapping techniques give quantitative measurements of extracellular volume (ECV) and provide an early marker of remodeling [31]. This has proven particularly useful in hypertrophic and restrictive cardiomyopathy, where diffuse fibrosis may occur before functional decline [32].

With the integration of the two techniques, strain echocardiography coupled with parametric mapping will allow clinicians to modify prognostic assessments and individualize treatment strategies; thus, these procedures are becoming more relevant in managing cardiomyopathy in modern times [33].

Myocardial work, described by Russell *et al*. [34], is an emerging echocardiographic tool that integrates left ventricular afterload into the analysis of global longitudinal strain. It has been studied across various clinical conditions to assess its added value compared to traditional metrics like left ventricular ejection fraction and global longitudinal strain. By enhancing the detection of subclinical cardiac dysfunction, myocardial work could serve as a valuable surrogate marker for disease, deepen our understanding of cardiac pathophysiology, guide in identifying therapeutic targets, and facilitate earlier diagnosis [35].

2.3.1.2 Genetics and Molecular Advances in Cardiomyopathy

Recent developments in gene-editing therapies, especially *CRISPR-Cas9* and RNA interference, promise to modify pathological genetic variants responsible for inherited cardiomyopathies [36]. These technologies are geared toward restoring the normal function of mutations in genes coding for sarcomere proteins involved in hypertrophic and dilated cardiomyopathies. In addition, genetic risk stratification using polygenic risk scores offers early detection and personalized management of cardiomyopathies, specifically in individuals with a family history.

A few new biomarkers have emerged as potential disease progression and prognosis predictors. Circulating miRNAs have shown promise for differentiating cardiomyopathy subtypes, whereas cardiac troponins have remained the most common markers for heart failure progression. Integrating multi-omics approaches, including proteomics and metabolomics, ought to refine risk prediction and facilitate targeted interventions [37].

#### 2.3.2 Imaging Examination

These imaging studies provide vital details about the anatomy and physiology of the heart, which are invaluable for diagnosing, classifying, and therapeutically assessing myocardial diseases. Generally, most clinical cardiologists choose imaging modalities based on the presentation and symptoms of an individual patient. Other technologies, such as imaging aided by artificial intelligence and quantitative imaging, have been introduced to expand diagnostic precision, especially in difficult cases of cardiomyopathy.

What are the most common imaging techniques used for diagnosing myocardial diseases? These techniques are described below in Table 1, with some advantages of each imaging modality around different types of cardiomyopathy diagnosis:

(1) Echocardiography:

Echocardiography is a method for cardiac structure and function assessment. It evaluates the dimension, shape, ventricular hypertrophy, wall motion, valve function, and other aspects of the heart. It is of great value for diagnosing and assessing DCM and HCM. Echocardiography is especially helpful in assessing ventricular dilation and decreased systolic function in DCM to identify asymmetric septal hypertrophy and assess left ventricular outflow obstruction in HCM [38].

AI-assisted echocardiograms are also coming out to be very helpful for certain automatic measurements like ventricular volumes and wall thickness estimation, which in turn may help early diagnosis and monitor disease progression.

(2) MRI:

Noninvasive cardiac MRI takes high-resolution images of the heart and myocardial tissue. MRI assesses ventricular wall thickness, heart capacity, and cardiac morphology and provides detailed myocardial function and hemodynamic information. It is useful in diagnosing and evaluating DCM and RCM, providing insight into cases with ventricular dilation + impaired systolic function. It gives information about myocardial fibrosis and tissue characterization, which is useful for managing HCM, followed by identifying the areas of myocardial scarring [39].

(3) CT:

The cardiac CT scan provides high-resolution images of the cardiac structure and lumen. It helps assess the anatomical structure and abnormalities of the heart and the cardiovascular system. This modality is extremely useful for diagnosing coronary artery disease (CAD) that may coexist with cardiomyopathy or in patients with complex structural defects. Although CT does not provide tissue characterization as MRI does, it remains the most valuable tool in assessing ventricular volume and left ventricular function in cases of DCM [40].

(4) CMR:

Magnetic resonance imaging with cardiac-magnetic resonance provides enhanced cardiac chambers and myocardium scans and much-needed detailed information on myocardial tissue. CMR plays an important role in the assessment of HCM and allows for the accurate identification of fibrotic tissue while distinguishing between hypertrophic and non hypertrophic myocardium. CMR can also detect myocardial scarring in DCM with accompanying left ventricular (LV) dysfunction, which plays a crucial role in RCM by assessing ventricle stiffness and diastolic function [41].

**Table 1.** **The common imaging inspection methods for myocardial disease**.

Inspection method	Inspection content	Evaluation content
Echocardiography	To examine the structure and function of the heart.	To assess the size, shape, ventricular hypertrophy, ventricular wall motion, valve function, and other aspects of the heart.
Magnetic resonance imaging	To provide high-resolution imaging of the structure of the heart and myocardial tissue.	To assess ventricular thickness, cardiac capacity, cardiac morphology, and can provide detailed information about myocardial function and hemodynamics.
Cardiac computed tomography	To provide high-resolution images of the cardiac structure and lumen.	To evaluate the anatomical structure and abnormalities of the heart and cardiovascular system.
Cardiac magnetic resonance imaging	Combined with MRI technology, can provide enhanced scans of the cardiac chambers and myocardium.	To offer more detailed information about myocardial tissue.

MRI, magnetic resonance imaging.

#### 2.3.3 Laboratory Inspection

Laboratory tests yield critical details to physicians about myocardial injury, inflammation, and cardiac function that help to diagnose, classify, and plan treatment for myocardial disease. These tests essentially guide acute management and long-term monitoring in patients with cardiomyopathy. The following are among the more commonly conducted laboratory tests for myocardial disease:

(1) Blood biochemistry indicators include troponin, creatine kinase isoenzymes, and myoglobin to assess myocardial cell damage and cardiac function. Raised troponin values are particularly useful for detecting acute myocardial injury or decompensations of dilated cardiomyopathy [38].

(2) B-type natriuretic peptide (BNP) or N-terminal (NT)-proBNP: BNP and its precursor N-terminal pro B-type natriuretic peptide (NT-proBNP) help to diagnose and monitor heart failure by providing information about pressure within the heart’s chambers and overall cardiac performance. NT-proBNP is particularly beneficial for identifying heart failure among RCM and DCM patients and can be useful in distinguishing between cardiac and non-cardiac causes of dyspnea [39].

(3) Complete blood cell count: This test assists in assessing myocardial inflammation and metabolic status by providing information on anemia, white blood cell count, and platelet count. Raised white blood cell counts could point to inflammatory causes of cardiomyopathy, such as myocarditis or infection-induced cardiomyopathy [40].

(4) Blood electrolyte tests: Tests include sodium, potassium, calcium, and several other electrolytes, assessing the electrophysiological function of cardiac cell electrolytes. Balance in the level of electrolytes is crucial, as an imbalance can induce arrhythmias, particularly in conditions such as hypertrophic cardiomyopathy or chemotherapy-induced cardiomyopathy.

(5) C-reactive protein: C-reactive protein is valuable for evaluating the systemic inflammatory response, including lesions in the myocardium, which is particularly pertinent in inflammatory cardiomyopathies such as myocarditis or sarcoidosis-associated cardiomyopathy [41].

(6) Coagulation function tests: These include prothrombin time and activated partial thromboplastin time, which help assess the functioning state of the coagulation system to prevent complications. This becomes particularly important for patients with HCM, who might be at higher risk for thromboembolic events due to left atrial dilation and atrial fibrillation.

(7) Genetic testing: Genetic testing enables patients with genetic myocardial disease to gain insights into genetic disease risk and etiology. Recent advances in next-generation sequencing give thorough analysis opportunities to examine cardiomyopathy genes such as myosin, troponin, and titin, directing toward early diagnosis, prognosis, and treatment decisions in familial cardiomyopathies like HCM and DCM.

#### 2.3.4 Genetic Examination

Genetic testing has much to offer in diagnosing, prognosis, and managing cardiomyopathy, especially genetic forms. It provides insights for physicians about the genetic etiology and family history of patients, such as genetic disease risks, inheritance patterns, and therapeutic implications. Genetic testing is valuable for the diagnosis of familial cardiomyopathies, thereby aiding clinical decision-making and developing a personalized treatment plan. Genetic testing must be considered on a patient-by-patient basis in clinical practice, based on the patient’s family history and clinical symptoms. The common aspects of genetic testing for cardiomyopathy include the following:

(1) Gene mutation screening: Gene mutation screening in cardiomyopathy patients, such as mutations in the myosin and troponin genes, assures the possibility of pathogenic mutations. Among such patients, genetic tests become very important, mainly in HCM and DCM, wherein certain genetic mutations correlate with the disease’s progression and treatment options. With the advances in next-generation sequencing (NGS), there is a possibility of detecting many mutations simultaneously, which will have greater diagnostic utility and personalized management based on genetic profiles.

(2) Family investigation: Conducting a family history investigation is essential for establishing whether other members of the patient’s family have cardiomyopathy or heart disease to assess genetic risk. Genetic counseling in families is especially valuable, as in familial cardiomyopathies, early diagnosis and surveillance can quite literally be life-saving.

(3) Genetic counseling: For patients with a family history, genetic counseling assesses the genetic risks of the patient and the relatives. This is specifically important in HCM and DCM, where early interventions (e.g., implantable cardioverter defibrillator (ICD)) may sometimes need to be guided by genetic findings. Genetic counseling also plays a major role in coping emotionally and psychologically with such knowledge.

(4) Family recurrence rate again assessment: Assessing the family recurrence rate allows for establishing an individual’s risk of having the disease. This is a resource to help conduct genetic screening of at-risk relatives. Families with an HCM or DCM history are often monitored closely to gain information from relatives who may be affected as early as possible so that appropriate preventive and early interventions may decrease morbidity and mortality.

#### 2.3.5 Emerging Treatment Strategies in Cardiomyopathy

The therapeutic landscape for cardiomyopathy has expanded by introducing novel therapeutics comprising pharmacologic and non-pharmacologic approaches. Sodium-glucose cotransporter-2 (SGLT2) inhibitors, which were developed originally for diabetes, have been shown to benefit patients with heart failure with preserved ejection fraction (HFpEF) and heart failure with reduced ejection fraction (HFrEF) by enhancing myocardial energy efficiency and reducing cardiac fibrosis [42]. HCM has a targeted therapy in the form of myosin inhibitors: mavacamten; these work by modulating sarcomere function to reduce left ventricular outflow tract obstruction and improve exercise tolerance. Furthermore, gene therapy trials investigate adenoviral vectors and antisense oligonucleotides to tackle pathogenic mutations associated with cardiomyopathies [43]. Taking all this into account, precision heading for managing cardiomyopathies with disease-modifying therapies likely extends beyond symptomatic relief.

### 2.4 Prognosis and Future Prospects of Cardiomyopathy

#### 2.4.1 Longitudinal Imaging and Biomarker Assessments

The longitudinal assessment of patients with cardiomyopathy has improved prognostic models for such patients. Serial echocardiography and cardiac MRI have become indispensable tools for monitoring remodeling of the myocardium, left ventricular function, and the progression of fibrotic changes over time [42, 44]. Such dynamic imaging modalities can provide ongoing insights into patient progress that static assessments at only one point in time miss, allowing for treatment tailoring based on real-time disease progression [14]. In addition, including serial biomarker measurements, such as NT-proBNP and troponins, in the longitudinal assessment of myocardial stress and injury enhances individual risk stratification [45]. Future studies should utilize AI-assisted serial analysis of imaging and biomarker trends to create refined predictive models for adverse cardiac events.

#### 2.4.2 Prognostic Factors

**Fig. 2.**
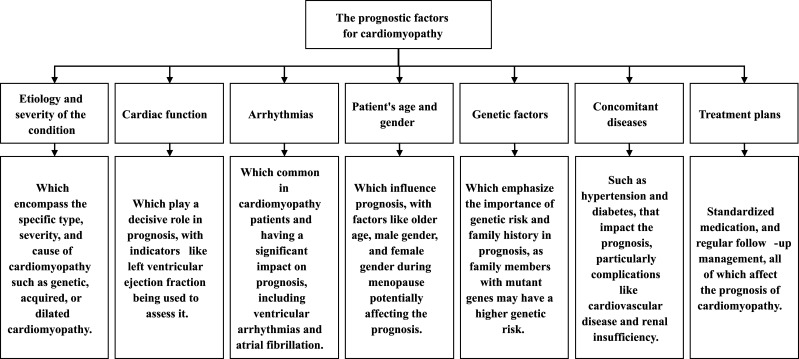
**The prognostic factors for cardiomyopathy**.

The prognosis of cardiomyopathy involves a variety of factors requiring the comprehensive assessment of the patient’s clinical profile, cardiac function, concurrent disease, and treatment plan [42, 43]. Critical aspects of prognostic evaluation are regular medical follow-up and assessment. Prognostic factors (Fig. 2) for cardiomyopathy mainly include: (1) Etiology and severity: The prognosis may depend on the specific condition, severity, and cause, e.g., genetic, acquired, DCM that has been proved to have an impact on prognosis. For example, genetic cardiomyopathies like HCM are highly variable in prognosis depending on the particular gene mutation. In contrast, acquired cardiomyopathies from myocarditis or chemotherapy-induced cardiomyopathy may exhibit more predictable disease behavior if the underlying cause is resolved in a timely fashion. Genetic testing advancements could offer more precise prognostic stratification for these disorders, allowing clinicians to predict disease progression and tailor treatment [14]. (2) Cardiac function: Cardiac function has been recognized as the most influential prognostic marker and is assessed through parameters such as left ventricular ejection fraction (LVEF). An LVEF drop in DCM means a bad prognosis, while, in HCM, patients may present normal to preserved LVEF; however, due to severe left ventricular hypertrophy (LVH) and outflow obstruction, they could be at an increased risk for arrhythmias and sudden cardiac death [45]. Recent advances, including cardiac MRI, are set to provide more detail on the level of myocardial fibrosis and tissue remodeling, which are associated with a poorer prognosis, particularly in HCM and DCM cases. (3) Arrhythmias: Arrhythmias are quite prevalent in cardiomyopathy patients and affect the prognosis significantly. Arrhythmias that occur with great incidences, especially ventricular arrhythmias and atrial fibrillation, are much higher in frequency in DCM and HCM, and their involvement can influence sudden cardiac death (SCD) risk. ICD usage is often considered appropriate in those at risk for high arrhythmias. Patients with a high risk for arrhythmias can also be identified with genetic testing to determine management strategies such as ICD implantation or antiarrhythmic therapy. (4) Age and sex: Patient characteristics affecting the prognosis include age and sex. Older age and male sex come with more risk for worse prognosis, whereas it is female sex and the menopausal period that also worsen prognosis and put patients at an increased risk for heart failure and cardiac remodeling. Age-related left ventricular function would complicate the disease prognosis; in elderly patients with HCM or DCM, comorbidities like hypertension and diabetes may hamper heart function. (5) Genetic factors: Genetic factors hold increasing importance in predicting the prognosis. Family history and genetic testing have shown that they help identify patients with high genetic risks for developing hereditary cardiomyopathies, including HCM, DCM, and arrhythmogenic right ventricular cardiomyopathy (ARVC). Knowing such gene mutations allows the clinician to predict disease prognosis and possible complications and determine targeted therapies [16, 46]. Genetic counseling is important for families that have a history of inherited cardiomyopathies because it will help them understand the risks to relatives to tailor appropriate screening. (6) Concomitant diseases: Concomitant diseases like hypertension, diabetes, and renal insufficiency could aggravate the prognosis of cardiomyopathies. Hypertension further increases the workload of an already affected heart, thereby contributing to ventricular hypertrophy development in HCM. At the same time, diabetes causes a more accelerated process of myocardial fibrosis and vascular damage in DCM. Chronic kidney disease, in some cases, represents another burden in managing cardiomyopathy in that it can further worsen fluid retention and heart failure. Therefore, management and treatment of these comorbidities can enhance the overall prognosis. (7) Treatment options: Treatment strategies, including medication, interventional procedures, and lifestyle changes, are paramount to the prognosis of cardiomyopathy. For example, beta-blockers and calcium channel blockers may palliate the symptoms of HCM and DCM, while angiotensin-converting enzyme (ACE) inhibitors and angiotensin receptor blockers (ARBs) may confer standard improvement in cardiac output in DCM. Heart transplantation is reserved for end-stage failures, particularly for patients with DCM or RCM who have not improved clinically with drug therapy. Patients require further monitoring for progression of disease, medication titration, and treatment of secondary complications due to arrhythmias or decompensation for the rest of their lives.

#### 2.4.3 Future Research Prospects

Research on heart diseases is bound to converge toward precision medicine and personalized treatment. This automatic combination of broader interdisciplinary approaches will include clinical medicine, bioinformatics, bioengineering, and further technology advances that will create a vision of timely developing more precise diagnostic and therapeutic means to ameliorate patient outcomes. In the years to come, cardiac disease research is fast moving towards areas such as (1) Efficient diagnostic techniques and novel biomarkers: The development of approaches to improve conditions for myocardial disease diagnosis to improve the early detection rates will be crucial. With the proper mix of genomics, proteomics, metabolomics, and AI tools, novel biomarkers can possibly account for improved diagnostic sensitivity. The initial results from applying AI algorithms to echocardiograms and cardiac MRIs show promise for automating diagnosis and predicting the course of the illness. In addition, an early understanding of biomarkers linked to particular types of cardiomyopathy would allow earlier and more directed intervention in certain cases, such as HCM and DCM. (2) Personalized treatment: Future therapies for cardiomyopathy will be designed to deliver personalized medicine. The possible treatments, such as gene therapy, gene editing, or stem cell therapy, may find excellent applications based on the genetic profile of the patient and related pathological characteristics. Genetic mutation-based targeted therapy for other cardiac genetic diseases, such as HCM or DCM, would improve patient quality by slowing progression and ensuring better treatment. Pharmacogenomics, addressing the gene influence upon drug response, will also contribute significantly to individualizing therapy among patients, thus minimizing adverse drug reactions and optimizing treatment effectiveness. (3) Imaging techniques: These techniques would revolutionize how one would work with cardiomyopathies regarding diagnosis and monitoring. Quantitative cardiac MRI allows key functional measurements during disease analysis, for example, at any given regional chamber of the beat, to measure their respective ventricular volume, myocardial strain, and fibrosis volumes. Quantitative echocardiography is still expected to assess left ventricular function indirectly and left ventricular hypertrophy. The depth of modality may as well allow improved results, sending it through various freshly developed AI algorithms with regards to automating the analysis of images, predicting disease outcomes, and, indeed, detecting early disease. Extended monitoring will now be part of the care oversight, with wearable devices displaying real-time monitoring and commensurate data for treatment tailoring opportunities. (4) New drug therapies: The future of myocardial disease treatment will also be creating new drugs specifically targeting the pathophysiological pathway implicated in disease status. Approaches that inhibit myocardial hypertrophy and fibrosis and improve dysregulated growing cells with current studies under microscopes are emerging today. Gene-based therapies focused on RNA interference and *CRISPR/Cas9* potentially enable changes in the underlying genetic mutations in hereditary cardiomyopathies that keep genes from being expressed and instead prevent them from exerting their harmful effects upon function. The development of heart failure drugs to restore cardiac remodeling will also slow disease progression and decrease HCM and DCM. (5) A genetic etiology study aims to analyze further by giving insight into the early detection, prevention, and treatment of myocardial diseases. Advanced next-generation sequencing technology will allow comprehensive genetic screening that should enable the identification of novel mutations causally related to cardiomyopathy. The functional understanding of the kinds of mutations and transmission pathways will help set the prognosis for disease onset in asymptomatic carriers of familial cardiomyopathies. Genetic counseling and early genetic screening will play very crucial roles in risk management and initiation of preventive care for families with a known history of cardiomyopathies. (6) Biomedical engineering in regenerative medicine: Biomedical engineering and regenerative medicine promise to offer a major impetus toward treating myocardial diseases, especially for repairing damaged myocardium. Techniques like myocardial tissue engineering and stem cell therapy are being developed with the potentiality of regenerating cardiac tissue, particularly in patients with end-stage heart failure. Developing organ-on-chip models and engineering approaches will permit the realization of patient-specific models for drug testing and regenerative treatment research. Using stem cells to repair damaged myocardial cells gives hope for reversing such damage, as seen in DCM and HCM.

## 3. Conclusion

Cardiomyopathy is a cardinal disease resulting from non-affectionate structural or functional anomalies of heart muscle, with a global prevalence. With an improved understanding of the etiology and pathogenesis of diseases, the diagnosis and classification of these diseases have also increased in sophistication. Nonetheless, reliability issues remain in their diagnostic techniques or prognostic models for much of the work done already, especially for genetic testing and imaging.

The impact of cardiomyopathy on cardiac function and quality of life is extreme and could lead to heart failure or premature death. A more definite grasp of disease mechanisms and early detection methods is needed to address these issues. This review does not just mention some highlights of the existing knowledge; it also discusses emerging trends in the diagnosis and treatment of cardiomyopathies, including integrating AI in imaging and genetic findings to personalize treatment strategies better. Ultimately, applying treatments based on genetic profiling and advanced diagnostics could yield much higher outcomes for these patients and reduce morbidity.

There will be considerable emphasis on developing individualized procedures in the management between now and the coming years of cardiomyopathy, where bioinformatics, bioengineering, multi-omic, and genomic research all come as one, providing integrative knowledge into the pathophysiological mechanisms of these types. New novel markers of injury, innovative imaging strategies, and targeted therapies will pave the way for novel innovations in diagnostics and therapeutics that will lead to much-needed prognostic accuracy, greater survival, and life for such patients with cardiomyopathy. Most importantly, these innovations will focus on early intervention and prevention, a more pre-emptive approach to managing cardiomyopathy and other allied heart diseases.

## Abbreviations

AI, Artificial intelligence; BNP, B-type natriuretic peptide; CRISPR/Cas9, Clustered regularly interspaced short palindromic repeats/CRISPR-associated protein 9; CRP, C-reactive protein; DCM, Dilated cardiomyopathy; HCM, Hypertrophic cardiomyopathy; ICD, Implantable cardioverter defibrillator; ICU, Intensive care unit; LVEF, Left ventricular ejection fraction; MRI, Magnetic resonance imaging; NT-proBNP, N-terminal pro B-type natriuretic peptide; NGS, Next-generation sequencing; RCM, Restrictive cardiomyopathy; SCD, Sudden cardiac death.
